# Accuracy and Fit of Three-Unit Dental Restorations Fabricated from 3D-Printed Resins and CAD/CAM Milling Materials: A Micro-CT Study

**DOI:** 10.3390/bioengineering13030362

**Published:** 2026-03-19

**Authors:** Jamila Yassine, Almira Ada Diken Türksayar, Florian Beuer, Nursena Öztemel, Franziska Schmidt

**Affiliations:** 1 Department of Prosthodontics, Geriatric Dentistry and Craniomandibular Disorders, Charité—Universitätsmedizin Berlin, Aßmannshauser Str. 4–6, 14197 Berlin, Germany; jamila.yassine@charite.de (J.Y.); florian.beuer@charite.de (F.B.); 2 Department of Prosthodontics, Faculty of Dentistry, Biruni University, 34015 Istanbul, Turkey; aturksayar@biruni.edu.tr (A.A.D.T.); nursenaoztemel@hotmail.com (N.Ö.); 3 Department of Advanced Digital Dentistry, Biruni University Advanced Technology and Research Center (BAMER), 34015 Istanbul, Turkey

**Keywords:** 3D printing, marginal adaptation, internal fit, micro-CT imaging, fixed dental prosthesis (FDPs)

## Abstract

(1) Purpose: To compare the fabrication accuracy, internal fit, and marginal adaptation of three-unit definitive resin fixed dental prostheses (FDPs) produced by subtractive milling and additive manufacturing. (2) Materials and Methods: A typodont mandible was prepared for a three-unit FDP, with full crown preparations on teeth mandibular left canine and mandibular left second premolar featuring 1 mm chamfer finish lines. The FDP was designed with a 16 mm^2^ connector and a 100 µm cement gap. Two milling materials (Ambarino High-Class, IPS e.max CAD) and two experimental 3D printing hybrid resins (3D-1, 3D-2) were used. All restorations were scanned using an intraoral scanner and compared to the original STL using reverse engineering software for surface trueness and deviation analysis. The internal fit was evaluated using the triple-scan method, while marginal fit was assessed via micro-CT imaging. Statistical analysis was conducted using one-way ANOVA and Kruskal–Wallis tests (α = 0.05). (3) Results: Milled groups demonstrated a lower prevalence of external, marginal, and overall surface deviations (*p* < 0.001), while 3D-1 exhibited comparable deviations within the internal region with M-E (*p* = 0.067). Milled groups had average gap values that were similar to 3D-1 (*p* > 0.08), but significantly lower than 3D-2 (*p* < 0.002). In marginal adaptation evaluated by micro-CT, the M-A and M-E groups provided significantly lower gaps, while the 3D-1 and 3D-2 groups exhibited wider marginal and axial gaps. (4) Conclusions: These results indicate that while milling remains a more reliable manufacturing method for achieving external and marginal precision, position 3D-1 is a compelling, chairside alternative to milling.

## 1. Introduction

There is an increasing interest in digital dentistry, and additive manufacturing technologies are evolving rapidly, while subtractive methods have become well-established in clinical practice [1,2]. However, subtractive manufacturing systems, also known as CNC milling methods, may not achieve the desired level of detail in machining due to the limitation in angulation capability and inability to reproduce complex internal or hollow geometries of the milling bur [3,4]. Additionally, material left over from blocks or disks cannot be reused [5]. Additive manufacturing technologies, which are based on the principle of layer-by-layer manufacturing, can produce even complex details with high precision and in a short amount of time [6,7]. Furthermore, even in chairside devices, it is possible to produce a large size of restorations at the same time [8,9].

According to ASTM/ISO (DIN EN ISO/ASTM 52900:2022 Standard Terminology [10] for Additive Manufacturing—General Principles and Terminology), additive manufacturing technologies are divided into the following seven different categories: vat photopolymerization, material extrusion, material jetting, binder jetting, powder bed fusion, directed energy deposition, and sheet lamination. Among these, vat photopolymerization is of particular relevance in dentistry because of its widespread use in the fabrication of resin-based materials. A wide range of resins, suitable for production through various vat photopolymerization methods, are employed by clinicians. Recent research has demonstrated the potential of these resins, which are often marketed for interim applications [11,12,13,14]. While new resin materials for permanent restorations have been introduced by different companies over time, these materials are only recommended for single-unit restorations [15,16,17]. It has been suggested that these resins, with different proportions and fillers, are comparable to hybrid ceramics used in subtractive fabrication [18,19,20,21,22]. Ceramic-filled resin materials for the fabrication of three-unit fixed dental prostheses (FDPs) are still novel, with no clinical and very little preclinical data available.

Achieving a precise fit in dental restorations is a fundamental challenge in prosthodontics [23,24]. Even small discrepancies can influence the longevity and functionality of fixed restorations. The clinically acceptable marginal gap is considered to be 120 µm [25]. Marginal gap (MG) and absolute marginal discrepancy (AMD) values above this threshold may lead to complications such as marginal leakage, secondary caries and periodontal problems [1,26]. Furthermore, discrepancies in different orientations can cause stress concentrations, which can compromise the mechanical stability and longevity of the restoration [16]. Methods such as silicone replica, cross section and triple scan are frequently used to evaluate the internal and marginal fit of restorations [17,27,28]. Among these, the silicone replica technique is a well-established alternative for assessing both internal and marginal adaptation. However, the triple scan method enables non-destructive three-dimensional analysis and a standardized digital workflow, which may provide more reproducible and less operator-dependent measurements.

In order to obtain precise and non-destructive measurements in the assessment of marginal fit, micro-computed tomography (micro-CT) is recommended, allowing a more comprehensive three-dimensional analysis compared to traditional two-dimensional assessment methods. Micro-CT provides detailed visualization of the internal and marginal spaces, facilitating a comprehensive assessment in all relevant anatomical directions [29,30].

A number of studies have previously evaluated the manufacturing accuracy, internal fit, and marginal compatibility of permanent resins used for different single-unit FDP. However, the available literature lacks sufficient data regarding permanent 3D printing resins that have been introduced for use in three-unit FDPs. Therefore, the aim of this study was to evaluate the fabrication accuracy, internal fit and marginal fit of three-unit tooth-supported FDPs produced by subtractive and additive fabrication methods. The null hypothesis of the study was that the fabrication method would not affect the fabrication trueness, internal fit and marginal fit.

## 2. Materials and Methods

A typodont mandibular model was prepared to replicate the missing mandibular left first premolar. A full crown preparation with a 1 mm thick chamfer finish line was made on mandibular left canine and mandibular left second premolar. The prepared model was then digitized using a 3Shape D2000 laboratory scanner (3Shape A/S, Copenhagen, Denmark). The exported file in standard tessellation language (STL) format was transferred to the dental design program. A three-unit fixed dental prosthesis was then designed, with a 16 mm^2^ connector thickness and a 100 µm cement gap, in accordance with the company recommendations. The occlusal thickness was set at 1.2 mm, while the axial thickness was fixed at 1 mm. The sample size was calculated based on a previous study 31 using software (G* Power, ver. 3.1.9.7; Heinrich-Heine-Universität Düsseldorf, Düsseldorf, Germany) that indicated 9 specimens per group (α = 0.05; power (1 − β) = 0.95; and f = 0.747). However, ten crowns per material were produced to enhance the statistical power (n = 10). Two different milling groups [hybrid material Ambarino High-Class (Creamed, Marburg, Germany; abbreviation M-A) and glass ceramic IPS e.max CAD, (Ivoclar Vivadent, Schaan, Liechtenstein; abbreviation M-E)] and two different experimental 3D printing materials [a ceramic filled resin for up to 3-unit FDPs (3D-1), and a ceramic filled resin for single restorations (3D-2), same company] were utilized in the study (Table 1). The milling of the blocks was conducted using a 5-axis milling device (K5, vhf camfacture, Ammerbuch, Germany). IPS e.max CAD samples were subsequently subjected to crystallization in a special furnace (Programat P100, Ivoclar Vivadent, Schaan, Liechtenstein). For the additive manufacturing groups, the same STL file was transferred to a nesting software program (Autodesk Netfabb Standard 2023, Autodesk, San Francisco, CA, USA). The specimens were positioned in a 45 degree angle on the build platform, in accordance with the manufacturer’s recommendation, and the supports were automatically positioned. The layer thickness was determined to be 50 µm. The design was then imported into the DLP (3D) printer (3Demax, DMG, Hamburg, Germany). The printed restorations were then subjected to a 3 min pre-cleaning and 3 min cleaning procedure with 96% isopropyl alcohol. Thereafter, the supports were removed and the restorations were cured in a xenon polymerization unit (Otoflash G171; NK Optik, Baierbrunn, Germany) with air contact under 2000 flashes, repeated twice.

All specimens from the production were stored in dry and lightproof boxes and then digitized with an intraoral scanner (CEREC Primescan SW 5.2; Dentsply Sirona, Bensheim, Germany). All specimens were scanned by the same experienced researcher. To test the production accuracy, each STL obtained was superimposed on the main production STL file using a reverse engineering program (Geomagic Control X 2022; 3D Systems, Rock Hill, SC, USA). The main production STL was selected as the reference and automatically segmented as external, internal, marginal and overall using the “region tool”. Each scan file was defined as measured data and the results were recorded as root mean square using the initial alignment and best fit alignment tools for superimposition, respectively. The “3D Compare” tool was used for color mapping. The area shown in green demonstrated acceptable deviation, and the tolerance range was determined as ±10 µm [17,31,32]. Red indicates overcontoured areas and blue indicates undercontoured areas.

**Figure 1:**
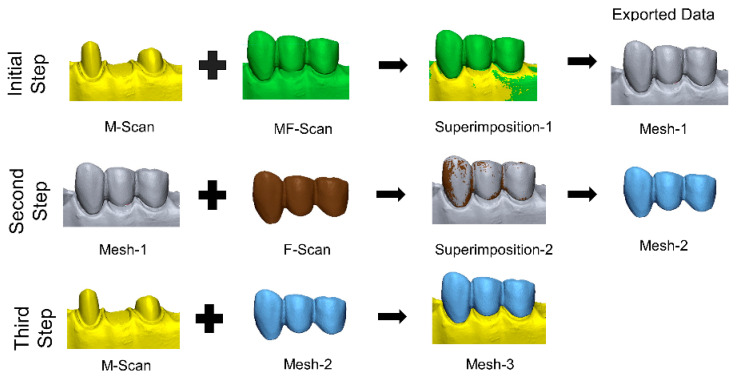
Workflow of triple scan protocol. M-Scan, master model scan; MF-Scan: fixed partial denture on master model scan; F-Scan: fixed partial denture scan.

The internal fit of the restorations was evaluated using the triple scan method (Figure 1), as previously described [17,32]. In the first step, the MF scan was superimposed onto the M-scan using the initial and best-fit alignment algorithms of the software program. This aligned dataset was exported as Mesh-1. In the second step, the F-scan was superimposed onto Mesh-1 using the same alignment procedure and exported as Mesh-2. In the final step, Mesh-2 was superimposed onto the M-scan to obtain Mesh-3, in which all datasets were aligned within the same coordinate system. Subsequently, section planes were generated in the buccolingual and mesiodistal directions for each prepared tooth at intervals of 0.3 mm. The distance between the preparation surface and the intaglio surface of the restoration was calculated using the Multiple 2D comparison tool.

**Figure 2:**
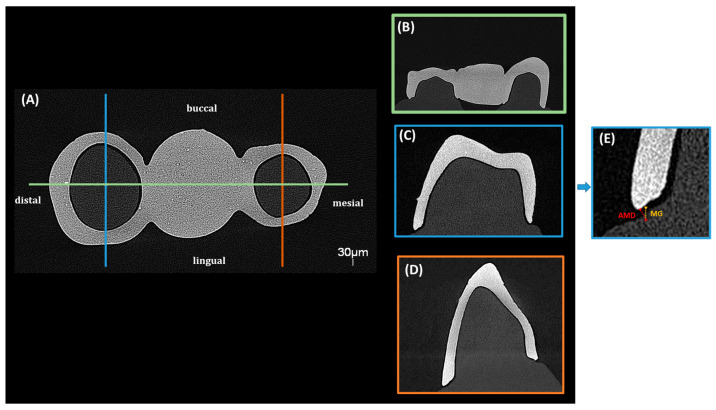
Micro-CT analysis of a 3D-printed three-unit bridge showing (**A**) coronal with underlying internal structure and sagittal sections for assessment, (**B**) mesial–distal, (**C**) buccal–lingual adaptation of the distal region and (**D**) buccal-lingual adaptation of the mesial region, with (**E**) magnified views illustrating marginal gap (MG) and absolute marginal discrepancy (AMD) at the bridge–model interface.

**Figure 3:**
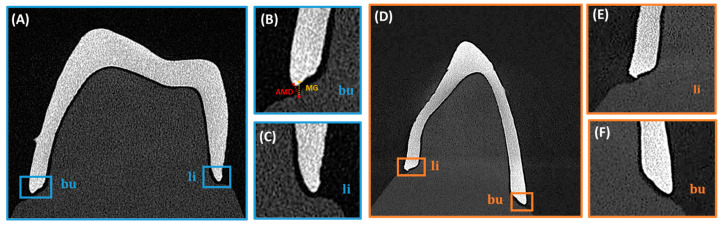
Sagittal sections of the (**A**) mandibular left canine showing (**B**) buccal (bu) and (**C**) lingual (li) aspects with magnified views for evaluation of marginal fit, marginal gap (MG), and absolute marginal discrepancy (AMD) and (**D**) of the second premolar with magnified (**E**) buccal (bu) and (**F**) lingual (li) aspects.

**Figure 4:**
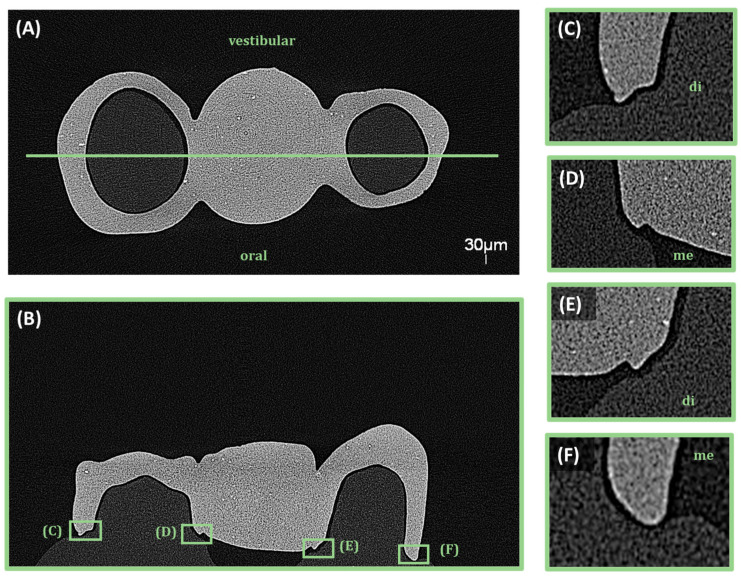
Coronal sections of the 3D-printed bridge showing the predefined mesial (me) and distal (di) measurement points at the contacts with the supporting teeth. (**A**) Coronal overview, (**B**) coronal section with contact points 5 distal, 5 mesial, 3 distal and 3 mesial, (**C**–**F**) high magnification of each contact point.

The fitting accuracy and MG measurements of the 3D-printed dental bridges were evaluated using a micro-computed tomography system (EasyTom S, RX Solutions, Chavanod, France). The scans were conducted with a voxel size of 30 µm, an accelerating voltage of 90 kV, a current of 333 µA, and a power of 30 W. A 0.3 mm copper filter was applied to reduce beam hardening artifacts and improve image contrast. The obtained volumetric data were reconstructed and evaluated using X-Act software (version 22.11.1, RX Solutions, Chavanod, France). To ensure a comprehensive assessment of the adaptation and gap distribution, specific sectioning planes were defined to capture relevant measurement points. Two main sectional orientations were analyzed (Figure 2, Figure 3 and Figure 4), sagittal and coronal. For sagittal sections the slices were obtained in the buccal and lingual regions, focusing on the interface between the restoration and the underlying model. The MG and AMD values were measured at predefined points along the span of the restoration to evaluate fit uniformity and adaptation. For coronal sections, they were positioned mesial and distal to the central axis of the bridge, assessing marginal fit at critical contact points with the model. The predefined measurement locations targeted areas most susceptible to discrepancies to ensure precise adaptation analysis.

The magnified sectional views provided high-accuracy measurements of MG and AMD values at the selected reference points. Segmentation and analysis were performed using threshold-based segmentation and manual calibration within X-Act software.

Statistical analysis was performed using statistical software (SPSS version 26; IBM Corp., Armonk, NY, USA). The normal distribution of the data was assessed with the Shapiro–Wilk test, and the homogeneity was assessed with the Levene test. Since all the data were normally distributed for trueness and internal fit, they were analyzed with one-way ANOVA. The Tukey post hoc test was performed for external surface trueness, internal fit, MG and AMD, while Games Howell post hoc test was performed for marginal, overall and internal trueness. Non-normally distributed micro-CT measurements were evaluated using the Kruskal–Wallis H test, and statistically significant differences were further examined by Bonferroni-corrected pairwise comparisons (α < 0.05).

## 3. Results

Trueness analysis of the external region revealed significant differences between the different groups (*p* < 0.001; Table 2). The highest RMS values were observed in the 3D-2 group (81.44 ± 9.59 µm), while the lowest were found in the M-E group (28.04 ± 5.87 µm). The 3D-2 group should have significantly higher deviations than all other groups (*p* < 0.001). The 3D-1 group also differed significantly from M-A (*p* = 0.003) and M-E (*p* < 0.001).

In the marginal region, significant differences were observed among the groups (*p* < 0.001). M-A showed the lowest deviation values, without significant difference compared to M-E (*p* = 0.695). A significant difference was found between 3D and 2 and 3D-1 (*p* < 0.05). The 3D-1 group exhibited significantly higher deviations than all other groups (*p* ≤ 0.007), while no difference was found between M-E and 3D-2 (*p* = 0.127).

Overall region analysis demonstrated significant intergroup differences (*p* < 0.001), with the lowest RMS values in the M-E group (34.61 ± 3.46 µm). The highest values occurred in the 3D-1 group, which did not differ significantly from either 3D-2 (*p* = 0.967) or M-A (*p* = 0.700).

For the internal region, the lowest deviations were observed in the 3D-1 group (30.51 ± 5.54 µm), with no significant differences compared to M-A (*p* = 0.072) or M-E (*p* = 0.067). However, 3D-1 differed significantly from 3D to 2 (*p* = 0.008). No significant differences were found between 3D and 2 and M-A (*p* = 1.000) or M-E (*p* = 0.404).

Internal fit evaluation using the triple-scan method also revealed significant group differences (*p* < 0.001; Table 3). The lowest internal discrepancies were observed in the M-E group (207.80 ± 18.69 µm), with no significant differences compared to 3D-1 (224.40 ± 6.50 µm; *p* = 0.080) or M-A (214.80 ± 18.47 µm; *p* = 0.723). No significant difference was detected between the 3D-1 and 3D-2 groups (241.30 ± 12.72 µm; *p* = 0.073).

Micro-CT analysis demonstrated significant differences in marginal gap (MG) and absolute marginal discrepancy (AMD) across fabrication methods and anatomical locations (Table 4 and Table 5). For the mandibular left canine, MG differed significantly among all groups in every measurement direction (*p* < 0.001). Both subtractive manufacturing groups (M-A and M-E) showed lower MG and AMD values than the additively manufactured groups (3D-1 and 3D-2).

For the mandibular left second premolar, MG and AMD differed significantly among groups in the coronal mesial, coronal distal, and sagittal buccal directions (*p* ≤ 0.004), while no significant differences were found in the sagittal lingual direction (MG: *p* = 0.080; AMD: *p* = 0.237). The lowest MG and AMD values were consistently observed in the M-A group, whereas the highest values occurred in the 3D-1 group.

## 4. Discussion

Fabrication trueness, internal fit and marginal fit were affected by fabrication method as significant differences were observed at each surface evaluated. Therefore, the null hypothesis was rejected.

The M-E group had the lowest RMS values for external and overall surfaces. The 3D-2 group had the highest external and overall RMS values. Although the maximum mean difference among the groups was limited (21.9 µm), the color map analysis was considered valuable because it provided additional qualitative information regarding the spatial distribution and localization of discrepancies that may not be fully reflected by mean values alone. Therefore, the color maps were additionally evaluated to better interpret the spatial distribution of deviations. Blue was the predominant color in the 3D-2 and 3D-1 groups, indicating a tendency toward undercontouring in both 3D-printed materials. Although this trend may partly be related to the additive manufacturing process itself, material-dependent factors such as resin matrix composition, polymerization behavior, and filler characteristics may also have contributed to the observed deviations. Since detailed information regarding exact filler type and full filler loading was not available for all tested materials, the individual influence of these parameters could not be determined in the present study. Clinically, undercontouring in 3D-printed groups may result in light or open interproximal contacts, which could lead to esthetic concerns, food impaction, gingival irritation, and the need for chairside adjustment. This result is consistent with previous findings about additively manufactured restorations [17,31,33]. The red color indicates overcontoured regions and was visible on the outside of the M-A group. It can be assumed that the M-A group’s esthetics are poor due to overcontouring, requiring clinical adjustments with tighter internal fit. The maximum difference in RMS values at the internal surface was 8 µm. Evaluation of intaglio surfaces revealed red areas in all groups, suggesting necessary clinical adjustments. When marginal surface deviations were considered, the M-A group had the lowest RMS values with a predominantly green color. The 3D-1 group had undercontoured areas with only slight spot-like overcontouring, meaning this group would have thinner margins and be more prone to deformation.

**Figure 5:**
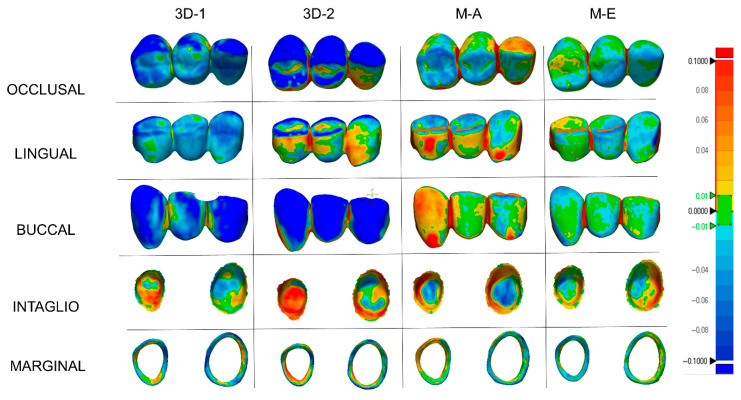
Representative color maps of tested groups for each surface.

Evaluation of the internal fit of the FDPs showed that the milled groups had a better fit than the 3D printed groups (Figure 5). Furthermore, the M-E group had the highest overall surface trueness while the lowest marginal RMS values were observed in the M-A group. Since marginal accuracy is closely associated with internal fit, these findings further support the overall outcome. Moreover, previous studies evaluated the accuracy of fit of milled and 3D-printed FDPs and concluded that the milled group showed a better fit compared to the 3D-printed group [32,34].

Additionally, a similar trend was observed when comparing the micro-CT findings to the triple-scan results. Both methods indicated superior adaptation in the milled groups M-E and M-A. However, micro-CT measurements yielded slightly higher discrepancy values compared to triple-scan data. This may be attributed to the superior spatial resolution and volumetric capabilities of micro-CT, which facilitate the detection of minor gaps and undercontoured regions that may not be fully captured by surface-based alignment methods.

Analysis of both MG and AMD across two teeth revealed consistent trends in material performance. The M-E and M-A groups demonstrated superior marginal adaptation, with significantly lower MG and AMD values and the most consistent fit across measurement sites. In contrast, 3D-1 exhibited the highest MG and AMD values, indicating inferior fit, particularly in the buccal direction, which may represent a technical challenge. The 3D-2 group often performed in an intermediate range, with variable results that were not always statistically different from the better-performing materials. The lingual direction showed fewer significant differences, suggesting a more uniform surface. A previous study assessed marginal adaptation using micro-CT and concluded that the 3D-printed group had superior adaptation [1]. The discrepancy with the present findings may be due to differences in study design, as the referenced study evaluated single crowns, whereas the current study assessed three-unit FDPs.

Research indicates that clinically acceptable marginal gaps for fixed restorations range from 50 to 200 µm, although many authors advocate for aiming for gaps of ≤120 µm to optimize long-term performance [35,36]. This threshold may also serve as a useful benchmark for interpreting results and comparing findings across future studies. The majority of MG and AMD values in the milled groups stayed well within the threshold, supporting their use. The 3D-printed groups had higher values in some regions, especially the buccal aspects, which might need adjustment during cementation. The 3D-printed restorations had higher values, especially in buccal regions. However, these differences may be partly because of the complex three-unit design and the limitations of the scanning strategy. This highlights the need for careful finishing and cementation to get the best fit in real cases. The FDP was placed in the area where the dental arch changes most and is not a straight line, so the internal fit may have been affected in these cases.

To the authors’ knowledge, no previous studies have evaluated the manufacturing accuracy, internal fit, and micro-CT-based marginal adaptation of M-A, a hybrid resin-based milling material with a filler content of 70.1 wt.%, in three-unit restorations. When comparing M-E, a lithium disilicate glass-ceramic, with the composite-based M-A, differences in marginal and external accuracy may be attributed to their fundamentally different material structures and machining behavior.

The observed differences in trueness and marginal adaptation between the materials may be partly explained by their distinct mechanical properties. M-E, a lithium disilicate glass-ceramic, exhibits a high elastic modulus and surface hardness, which may promote greater dimensional stability and edge fidelity during the milling process. In contrast, M-A, a composite-based CAD/CAM material with a substantially lower elastic modulus and hardness, may be more susceptible to deformation and reduced marginal precision, particularly in fine anatomical regions. Future studies should further investigate how the mechanical and physical properties of these materials, including elastic modulus and hardness, may influence marginal precision and overall clinical performance.

Only one 3D printing technology was used to produce the printed materials evaluated in this study. Differences in the selection of 3D printers or milling systems and changes in printing parameters could potentially affect the results. The type and amount of inorganic fillers may also influence the dimensional behavior of printed resin materials. According to the manufacturer-provided information, the inorganic filler content was 30–50 wt.% for one experimental resin and >55 wt.% for the other; however, the exact filler type and detailed filler composition were not disclosed. This limited compositional transparency should therefore be considered when interpreting the present findings. Furthermore, a currently established conventional resin-based material was not included as a comparator. Although its inclusion could have provided an additional clinical reference point, the present study was specifically designed to evaluate recently introduced ceramic-filled resin materials for three-unit FDP fabrication. Future studies should compare these materials with conventional resin-based alternatives to provide broader clinical context. Another limitation is the use of a three-unit FDP design between mandibular left canine and mandibular left second premolar, corresponding to the transition zone of the dental arch. This area represents a curvature shift between the anterior and posterior segments, which has the potential to complicate the scanning, design, and milling processes. Although the present study focused on the transition zone of the dental arch as a clinically demanding region, future studies should also investigate more favorable arch regions to allow broader comparison and improve generalizability.

Although the triple scanning method is efficient and reproducible for assessing internal consistency, it may not fully reflect localized volumetric differences as it relies on surface alignment. Micro-CT, on the other hand, can evaluate both internal and marginal adaptation in high-resolution 3D, but it is susceptible to artifacts, such as beam hardening, particularly in high-radiopaque materials [17]. Further studies directly comparing these two methods under standard conditions will provide more comprehensive information about their clinical applicability and accuracy.

## 5. Conclusions

Within the limitations of the present study, the following conclusions can be drawn:

1. The fabrication method had a significant impact on the trueness, internal fit, and marginal adaptation of the three-unit FDPs.
2. Milled restorations, particularly from lithium disilicate glass-ceramic (M-E), demonstrated superior performance in terms of surface trueness and internal fit.
3. While the additively manufactured groups demonstrated acceptable values, undercontouring was more evident, particularly in the 3D-2 material.
4. Micro-CT analysis revealed that marginal gaps in 3D-printed restorations, especially in buccal regions, were frequently higher compared to the milled groups and in some cases exceeded clinically recommended thresholds.

## Figures and Tables

**Table 1:** Used materials, processing method, and general composition.

Group Name	Material	Processing	General Composition
3D-1	Experimental hybrid resin material-1	3D printing	Acrylate resin with inorganic fillers (30–50 wt.%; exact filler type not disclosed by the manufacturer)
3D-2	Experimental hybrid resin material-2	3D printing	Acrylate resin with inorganic fillers (>55 wt.%; exact filler type not disclosed by the manufacturer)
M-A	Hybrid ceramic, Ambarino High-class, creamed GmbH, Marburg, Germany	Milling	Highly crosslinked Bis-GMA, UDMA, Butandiaol dimethacrylate, 70.1 wt.% anorganic silica nanofillers. Indications: inlays, onlays, crowns, FDP (max. 3-unit)
M-E	Glass-ceramic, IPS e.max CAD, Ivoclar Vivadent, Schaan, Liechtenstein	Milling	Lithium disilicate-reinforced glass-ceramic (70% lithium disilicate). Indications: inlays, onlays, crowns, FDP (max. 3-unit)

**Table 2:** Mean surface deviations and standard deviation values of the trueness given as mean RMS values (µm).

Group	Trueness (RMS) in µm			
	External	Marginal	Overall	Internal
3D-1	63.41 ± 6.48 ^c^	41.18 ± 4.53 ^c^	56.49 ± 5.49 ^b^	30.51 ± 5.54 ^a^
3D-2	81.44 ± 9.59 ^d^	34.90 ± 1.69 ^b^	54.83 ± 10.00 ^b^	38.12 ± 2.25 ^b^
M-A	48.36 ± 12.30 ^b^	28.51 ± 5.77 ^a^	52.50 ± 10.16 ^b^	38.28 ± 7.41 ^b^
M-E	28.04 ± 5.87 ^a^	31.09 ± 4.67 ^ab^	34.61 ± 3.46 ^a^	36.09 ± 3.31 ^ab^

Different superscript lowercase letters indicate significant differences among test groups in each column (*p* < 0.05).

**Table 3:** Mean ± standard deviation of average gap values (µm) derived from triple scan.

Group	Average Gap (µm)
3D-1	224.40 ± 6.50 ^ab^
3D-2	241.30 ± 12.72 ^b^
M-A	214.80 ± 18.47 ^a^
M-E	207.80 ± 18.69 ^a^

Different superscript lowercase letters indicate significant differences among test groups in each column (*p* < 0.05).

**Table 4:** Mean ± standard deviation of marginal gap (MG) and absolute marginal discrepancy (AMD) values [µm] for mandibular left canine.

Direction		3D-1	3D-2	M-A	M-E	*p* Value
MG	coronal mesial	149.23 ± 94.82 ^b^	153.54 ± 82.71 ^b^	72.88 ± 14.74 ^a^	66.04 ± 13.80 ^a^	*p* ≤ 0.001
	coronal distal	148.10 ± 93.04 ^b^	158.50 ± 81.56 ^b^	83.61 ± 16.68 ^a^	71.72 ± 20.18 ^a^	*p* ≤ 0.001
	sagittal buccal	189.47 ± 63.62 ^b^	176.00 ± 99.24 ^b^	75.61 ± 8.60 ^a^	117.76 ± 13.90 ^ab^	*p* ≤ 0.001
	sagittal lingual	115.08 ± 61.11 ^a^	230.40 ± 117.05 ^b^	130.68 ± 19.11 ^a^	188.90 ± 13.65 ^b^	*p* ≤ 0.001
AMD	coronal mesial	179.11 ± 84.05 ^b^	171.11 ± 91.14 ^b^	82.60 ± 16.08 ^a^	71.52 ± 17.69 ^a^	*p* ≤ 0.001
	coronal distal	159.68 ± 118.48 ^b^	156.80 ± 81.62 ^b^	88.35 ± 13.66 ^a^	75.76 ± 16.84 ^a^	*p* = 0.002
	sagittal buccal	198.30 ± 52.88 ^b^	177.49 ± 107.18 ^b^	86.38 ± 8.99 ^a^	127.02 ± 23.29 ^b^	*p* ≤ 0.001
	sagittal lingual	173.30 ± 115.57 ^b^	238.00 ± 65.13 ^b^	145.74 ± 21.56 ^a^	188.16 ± 73.42 ^b^	*p* = 0.004

Different superscript lowercase letters indicate significant differences among test groups in each row (*p* < 0.05).

**Table 5:** Mean ± standard deviation of marginal gap (MG) and absolute marginal discrepancy (AMD) values [µm] for mandibular left second premolar.

Direction		3D-1	3D-2	M-A	M-E	*p* Value
MG	coronal mesial	182.30 ± 133.81 ^a^	163.40 ± 76.44 ^a^	76.84 ± 11.80 ^b^	101.48 ± 11.20 ^ab^	*p* = 0.002
	coronal distal	134.87 ± 64.16 ^c^	92.30 ± 27.97 ^bc^	48.96 ± 5.03 ^a^	67.17 ± 13.04 ^ab^	*p* ≤ 0.001
	sagittal buccal	209.20 ± 56.42 ^c^	139.00 ± 32.19 ^bc^	73.18 ± 12.10 ^a^	97.80 ± 37.72 ^ab^	*p* ≤ 0.001
	sagittal lingual	164.78 ± 97.72 ^a^	149.90 ± 52.76 ^a^	130.41 ± 25.07 ^a^	167.90 ± 24.51 ^a^	*p* = 0.080
AMD	coronal mesial	193.39 ± 152.64 ^b^	181.40 ± 79.40 ^b^	89.70 ± 5.87 ^a^	110.36 ± 115.04 ^b^	*p* = 0.004
	coronal distal	135.01 ± 50.81 ^c^	116.77 ± 0.04 ^bc^	63.74 ± 7.15 ^a^	85.97 ± 40.54 ^ab^	*p* ≤ 0.001
	sagittal buccal	182.40 ± 57.17 ^b^	160.80 ± 50.26 ^b^	87.37 ± 8.73 ^a^	98.03 ± 23.80 ^a^	*p* ≤ 0.001
	sagittal lingual	155.29 ± 80.36 ^a^	167.20 ± 73.09 ^a^	147.60 ± 22.36 ^a^	172.80 ± 27.74 ^a^	*p* = 0.237

Different superscript lowercase letters indicate significant differences among test groups in each row (*p* < 0.05).

## Data Availability

The original contributions presented in this study are included in the article. Further inquiries can be directed to the corresponding author.
